# The need for assisted reproductive technology regulations: a case for women in the Philippines

**DOI:** 10.3389/fsoc.2025.1429980

**Published:** 2025-03-19

**Authors:** Hazel T. Biana

**Affiliations:** Southeast Asia Research Center and Hub, De La Salle University, Manila, Philippines

**Keywords:** *in vitro* fertilization, RH law, Philippines, infertility, reproductive rights

## Abstract

No laws regulate Assisted Reproductive Technology (ART) in the Philippines. Because of this, women who suffer from infertility must settle with specific guidelines crafted by medical and fertility specialists and professional organizations. As a result, women have limited access to ART and rely on scarce healthcare services and facilities, which may be at the mercy of several guidelines influenced by personal and religious beliefs. In this essay, I examine these regulations (or lack thereof), their socio-cultural motivations, and their dire implications on women and their reproductive rights. I show that Philippine ART regulations lag compared to some developing countries; women have limited choices to address their fertility and reproductive health issues, and they lack the support that they need in dealing with infertility. Thus, regulations need to be crafted to make ART practices more inclusive and less inhibiting for women in the Philippines.

## Introduction

*In vitro* fertilization (IVF) technology is one of the most common procedures used in assisted reproductive technologies (ART), wherein fertility-related issues are treated through medical interventions by manipulating gametes or embryos (Jain and Singh, 2025). The process involves the collecting oocytes from the ovary, fertilizing them *in vitro*, and transferring the resulting embryo into the uterus (Jain and Singh, 2025). Other associated techniques would also include cryopreservation and intracytoplasmic sperm injection (ICSI). The first successful IVF live birth occurred in the United Kingdom in 1978. In a developing country such as the Philippines, the first live IVF birth occurred in 1996 (Dupont, 2013).

Despite the availability of ART, however, many women and couples all over the world still struggle to conceive. In the Philippines alone, one out of four couples still deal with infertility issues (Flores, 2016). In Taguibao and Bance's study (2022, p. 32), for instance, some Filipino women shared that dealing with their fertility issues is an “uphill battle.” From the realities of treatment and diagnosis to the emotional pain brought about by unsuccessful procedures, women feel stressed and powerless (Taguibao and Bance, 2022, p. 32–33). Financial stress is also significant burden, especially when considering the costs of workups, medications, and other necessary resources. In perspective, one IVF cycle in the Philippines costs about half a million pesos (~10,000 USD). This estimate does not include the additional costs of medications required for the procedure. As such, some couples would resort to undergoing the procedure in other countries, such as Taiwan.

At present, centers in the country offer many ART services in addition to IVF. These include follicular monitoring, intrauterine insemination (IUI), ICSI, assisted hatching, cryopreservation, embryo transfer, blastocyst transfer and culture, and others. Unfortunately, infertility issues are not covered by health insurance in the country, so all these costs are shouldered out of pocket. Like the rest of the world, the question of who should have access to such technologies and who should pay for it is still being debated (Nugent, 2018). In the Philippines, the Republic Act No. 10354 or the Responsible Parenthood and Reproductive Health Law could have included provisions for infertility treatments and ART to support women and couples with fertility issues. Its coverage and implementation, however, is limited and hindered by the opposition of some ultra-conservative groups.

## The reproductive health law of the Philippines

The reproductive health law (RH Law), Republic Act No. 10354, or the Responsible Parenthood and Reproductive Health Act of 2012, is one of the most highly debated laws in the Philippines. With a predominantly Catholic population, several fundamental groups have questioned its constitutionality and fought to hamper its implementation. It is not surprising that what the government would consider as “groundbreaking” with this law is just mere access to contraceptives, information on family planning, reproductive health education, and women's rights to postabortion care. While developed countries would consider these provisions as fundamental human or health rights, the law's full implementation has yet to happen despite being passed more than 10 years ago.

The law defines reproductive health as “all matters relating to the reproductive system and its functions and processes.” Still, it does not comprehensively include fertility for its citizens' complete physical, mental, and social well being. Despite its mention of “infertility” twice, concerning the prevention, treatment, and management of infertility and sexual dysfunction and reasonable procedures for poor and marginalized couples, it does not specify these treatments at all. Furthermore, while reproductive health care is defined as access to a full range of methods, facilities, services, and supplies that contribute to reproductive health and well being by addressing reproductive health-related problems, it does not mention (ART) in its provisions.

## A lack of law for assisted reproductive technology

The RH Law has no provisions or support for ART methods or services such as IUI, IVF, and other reproductive health methods facilities, services, and supplies. This means that while the law seeks to provide citizens with reproductive health care, it is unclear on its provision for reasonable procedures when it comes to infertility. As such, there are two immediate implications of this omittance or the lack of a law on ART thereof; first, persons with fertility problems who require more advanced technologies are neglected in their quest for reproductive well being, and second, medical specialists and professional societies would self-regulate their guidelines and policies on ART. I argue that this non-regulation or lack of regulation results in Filipino women's deprivation of their reproductive autonomy and their inaccessibility to fertility treatments, which the World Health Organization (WHO) recognizes as essential medical healthcare (Hill, 2020).

The inaccessibility of fertility treatments and the existence of ART were introduced to policymaking by a female lawmaker in the country in 2007 and 2010. In the first regular session of the Fourteenth and Fifteenth Congress of the Republic of the Philippines, respectively, Senator Miriam Defensor Santiago filed versions of the Family Building Act, which requires coverage for infertility treatment in any group health plan or health insurance. This act acknowledges that millions of Filipino women and men are suffering from fertility issues. Considering that the majority of group health plans do not provide coverage for infertility treatments, the act recognizes the impossibility for low and middle-income families to avail of ART services. The act also defines infertility as a disease or condition of the reproductive system and ART as treatments or procedures such as IVF, gamete intrafallopian transfer, zygote intrafallopian transfer, embryo cryopreservation, egg or embryo donation, and surrogate birth.

The Family Building Act was so progressive that it was never ratified into law. Consequently, since the RH Law does not mention ART, no other law in the country regulates or mentions ART besides the Family Code, which recognizes artificial insemination. Likewise, there are no regulatory frameworks for egg freezing or surrogacy. Furthermore, while the Department of Health has rules and regulations governing “other” health facilities, such as specialized out-patient facilities like IVF services, no specific laws governing their establishments or licensing procedures exist for ART services (In-Vitro Fertilisation, Laws and Regulations, 2021). As such, medical specialists and professional organizations such as the Philippine Society for Reproductive Medicine (PSRM), the Philippine Society of Reproductive Endocrinology and Infertility (PSREI), and the Philippine Obstetrical and Gynecological Society (POGS) are left to craft their guidelines for lack of available regulation on ART, precisely the *Guidelines on the Ethics and Practice of Assisted Reproductive Technology and Intrauterine Insemination*. The trouble with privatizing such self-regulation is that policies may be motivated by non-altruistic moral preferences (Baron, 2010) and need more accountability in the event of regulatory failures (Priest, 1997).

One of the primary motivations of the RH Law is to ensure that reproductive healthcare is not refused to a person based on the healthcare provider's sociocultural views or the citizen's intersectional factors such as marital status, gender, etc. Such refusal is deemed prejudicial to women from low-income classes, especially those who rely on more modern family planning methods. Some examples of prohibitions in refusal include “voluntary ligation and vasectomy and other legal and medically-safe reproductive health care services” (Cabral, 2013). In the United States, for instance, Catholic hospitals prohibit sterilizations, but the ban is not enforced uniformly as some obstetrician-gynecologists are conflicted with such religiously motivated policies (Stulberg et al., 2014). Incidentally, there are many cases wherein ethical or religious views of healthcare providers become the basis for their refusal to provide care, since procedures are seen as anti-life or anti-family by some Philippine religious institutions and their practitioners (Chiu, 2012).

## Experiencing ART

With ART absent in the RH Law provisions, my struggles with infertility are my personal struggles alone. With no information or health support, I had to rely on women's online forums, friends, and the Internet. On top of the social pressure of having to conceive as a woman and the emotional and psychological stress, my consultations with fertility specialists were time-consuming and expensive. I could not take a sick day leave, and no private or public health insurance would cover obstetrician-gynecologists or specialists' consultations and fertility workups. The doctors would ask me if I was committed to “treating” my infertility and accounting for expenses that came with it. They would require various tests such as blood tests for hormonal levels (such as the follicle-stimulating hormone, luteinizing hormone, estradiol, and others), transvaginal ultrasound procedures (TVS), and the Hysterosalpingogram (HSG) (which is a test to check the fallopian tubes for possible blockages).

Committing to ART, I would undergo these blood tests and TVSs every week, and I would also take prescription medications to stimulate hormones and produce high quality eggs. I would undergo various ART interventions to conceive. These would include timed intercourse, intrauterine insemination (IUI), and eventually IVF. Since the fertility specialist was somewhat conservative, I had to start with the least aggressive and “cheaper” ART methods. Having to spend for these less-aggressive methods turned out to be more expensive in the long run. If we had gone straight to IVF, we would have had higher chances of conceiving with the least amount of stress, time and money. Proper information, guidance, and support would have been beneficial to women who have fertility challenges like me.

## Motivations of ART guidelines

With a lack of explicit governmental policy regarding ART services, I argue that moral and religious views have motivated ART providers' professional self-regulation and policies in the Philippines. In fact, some provisions in the guidelines are even labeled “pro-life” and “pro-marriage,” to insinuate their reflection of Catholic or Christian values (Chiong, 2022). The same goes with the RH Law, wherein it puts a premium on the freedom of conscience when it comes to decision-making concerning medical procedures and state-sponsored reproductive health programs. This means that “the state cannot compel any of its personnel to implement any contraceptive or reproductive health procedures that may be deemed unethical or immoral by them (based on their religious convictions)” (Lofredo, 2016, p. 332). As such, available reproductive health services depend highly on ethical and religious beliefs or convictions. Such conscientious objections, however, are deemed unethical as they can be abused to compel patients, specifically women, to adhere to spiritual or religious values they do not believe in (Dickens, 2006). Dickens (2006) believes that the only way conscientious objections may be “ethical” is if healthcare providers refer women to other providers and the government ensures access to these providers. Given the case of ART and the silence of the RH Law about it, one can infer two contradictory things: that the government must ensure access to fertility treatments or methods. Still, given the operational word “reasonable,” it does not need to provide access to more expensive ART treatments. Thus, one can ask whether conscience-based regulations on ART are ethical, given that the government is not mandated to ensure access to ART providers.

Without actual laws on ART in the country, the same subjective conscience-based rulemaking (which may be altruistic or not) is the basis of existing professional regulations on ART (Aguilar et al., 2024). One of these self-regulated guidelines includes the previous prohibition of preimplantation genetic screening (PGS) or the screening of embryos for any chromosomal abnormalities to help avoid natural abortion. As a result, Filipinos previously traveled to Taiwan or other countries to undergo such screening. While preimplantation genetic testing for aneuploidies (PGT-A) is available in some major ART centers in the country (Abad et al., 2023), this test does not cover the service usually requested by those with known hereditary diseases that may be passed on to their offspring. In 2016, preimplantation genetic diagnosis (PGD) and preimplantation genetic screening (PGS) have been deemed ethically acceptable in cases of genetically transmitted conditions “which are serious, and no safe and effective interventions are available (Chiong, 2022, p. 508).” Embryonic gender identification is also now allowable “in cases where a strong family history of sex-linked genetically transmissible disease exists (Chiong, 2022, p. 508).” If a case does not fall under “serious” or “strong family history,” then sex screening cannot be performed. Should a couple have other sex-linked genetic diseases, they would have to go elsewhere for such tests. As a result, Filipinos must rely on existing (possibly conscience-based) guidelines alone since there are few genetic counselors in local IVF centers (Abad et al., 2023). Such cases are confirmed by a study with predominantly Christian respondents which show the correlation between the decision to undergo PGS and the importance of religious beliefs and ethical values (Gebhart et al., 2016). Another study explicitly finds that accepting PGT as an antenatal option is decided by one's religion (Zuckerman et al., 2020).

Other “rules” for ART and IVF in the country include a maximum of two embryos allowed for transfer during a single procedure (Dupont, 2013). Kato Repro Biotech Center (Kato), a leading ART center in the Philippines, prefers “single embryo transfer to lessen the complications of multiple gestations” (FAQs | Kato Repro Biotech Center, 2025). The only time Kato allows the transfer of two embryos is when couples wish to increase their chances of having twins. In the US, there have been debates on whether the State should regulate the allowable number of embryos to be transferred. Some argue that limiting the number of embryos in one transfer goes against the right to procreate (Forman, 2011). In the Philippines, however, the RH Law explicitly states that:

“The State shall promote programs that: (1) enable individuals and couples to have the number of children they desire with due consideration to the health, particularly of women, and the resources available and affordable to them and in accordance with existing laws, public morals and their religious convictions: Provided, That no one shall be deprived, for economic reasons, of the rights to have children...”

If we were to follow this strictly, then the local professional regulation of multiple embryo transfers could be questioned. While such a guideline is for the safety and benefit of the mother and babies, the lack of mention of ART in the RH Law assumes that refusing multiple embryo transfers without explicit medical justification is against the law, specifically the right to have (the desired number of) children.

The most conservative and perhaps discriminatory ART guideline is that only legally married couples can undergo ART procedures in the Philippines. As such, third-party donor eggs, sperm, or embryos may not be used in the treatment. This means that unmarried couples, same-sex couples, or single people cannot avail of ART services in the country. This is quite ironic given that the Family Code of the Philippines acknowledges that a child born out of artificial insemination through donor sperm is a legitimate child of the husband and wife. This implies that the Family Code recognizes third-party artificial insemination despite its prohibition by professional regulation in the country. Such a guideline is discriminatory to marginalized members of Philippine society, such as single persons, LGBTQI aspiring parents/couples, and unmarried couples. One study that reviewed the implications of specific policies on lesbian single parents, for instance, claims that the Philippine medical ethics boards prevent single LGBTQIs from undergoing advanced fertility treatments (Biana and Domingo, 2021). Such policies may be traced to the Catholic Church's historical exclusion of non-heteronormative individuals and “those who do not conform to conservative gender constructs” (Biana et al., 2022).

The same prohibition for particular ART treatments applies to unmarried couples. Filipino single women will have to settle with freezing their eggs for future use if and when they decide to tie the knot with their male partners. A case study by Shirai (2021) even divulged that some Philippine Catholic hospitals have banned IVF and gamete and embryo freezing. Additionally, unmarried couples cannot undergo certain ART or IVF procedures that involve the meeting of gametes. In the Philippines, only *homologous* IVF is permitted. This means that the gametes used must come from legal spouses. This guideline is primarily and obviously motivated by religious convictions. Not being married and having children is, after all, anti-family, according to the Catholic Church. Conservative culture dictates that only married couples should have children. This guideline seems to go against the RH Law, particularly the refusal to “extend quality health care services and information on account of the person's marital status, gender, age, religious convictions, personal circumstances, or nature of work.” Then again, the RH Law has no mention of ART.

## The lack of support for women

Aside from the lack of access of marginalized individuals and non-traditional couples to several ART services, there is also a dearth of laws that support women undergoing fertility treatments. First off, there are no leaves provided for women who are undergoing ART treatments. One could argue that since infertility is a disease, perhaps sick leaves may be used for such a purpose. In the Philippines, however, employers are not legally required to offer sick leave days to their employees. While some provide around 12–15 sick days, workers must provide medical certificates to justify absences. While there is a 105-Day Expanded Maternity Leave Law in the country, such leave is prenatal and postnatal, meaning a woman should already be or have been pregnant before she can avail of it.

Some institutions in more developed nations, such as the University of Oxford, provide fertility treatment leaves on top of medical appointment leaves, sickness leaves (which can be used for physically recovering from treatment procedures or illnesses from fertility treatment or pregnancy), and pregnancy leaves (The People Department, 2023). Such benefits are also available to staff going through a surrogacy arrangement. While there are no specific statutory rights to attend IVF treatment in the United Kingdom, the additional leave addresses the discrimination and struggle suffered by women undergoing fertility treatment (Murray-Nevill, 2023). Women's rights activists argue that an inclusive workplace culture should provide infertility support to women (Skinner and Clark, 2021).

In the Philippines, the absence of workplace support for women undergoing fertility treatments, coupled with the lack of coverage from local health insurance providers, may lead to women discontinuing their treatment for various reasons beyond financial constraints. A study done in Belgium (Van den Broeck et al., 2009) shows that women discontinued their fertility treatments primarily due to their psychological and physical burdens. A lot of women even refused to be involved in the study because of the refusal to relieve the history of their infertility. When fertility treatments fail, women are vulnerable, helpless, and anxious, and they need acceptance and social support. Such burdens are attributed to emotional distress, stress, depression, the physical pain of injections, and other side effects (Gameiro et al., 2012). Mental health professionals recommend counseling for women who have failed or discontinued their ART treatments (Ebrahimzadeh et al., 2019).

The psychological and emotional support of women would have been supplemented by the Republic Act No. 11036: An Act Establishing a National Mental Health Policy for the Purpose of Enhancing the Delivery of Integrated Mental Health Services, Promoting and Protecting the Rights of Persons Utilizing Psychosocial Health Services, Appropriating Funds Therefor and Other Purposes. However, since there is no specific ART laws integrated into the RH law and infertility treatment is not considered an essential health service, the national mental health care system is not mandated to include specific mental health care stipulations for women undergoing infertility or fertility treatments in their essential mental health services and community-based facilities. While the act mentions the terms “gender-sensitive” and “responsive to gender,” it does not mention any gender-specific advocacies, treatments, or protocols concerning the type of psychosocial support for women or women undergoing ART at that.

## Implications

Aside from the reproductive healthcare providers' potential lack of accountability and government or community-specific support, the problem with the absence of regulatory laws on ART and IVF is that women's bodies and their reproductive health choices may be “regulated” by the moral or religious inclinations of medical specialists and professional societies. They may argue that they are objective experts in their field and that their ART regulations are altruistic and in line with Philippine socio-cultural values and norms. We should ask, however, whether the ART guidelines are liberal enough to accommodate women with several intersectional factors and particular reproductive and psycho-social needs. Kaplan (2024, p. 197), for instance, claims that women who experience infertility and pursue ART methods “require additional psychoeducation and support” since their decision may go against religious beliefs and teachings. Kaplan (2024, p. 197) further states that they would need critical sources of support and “aid in coping.”

Along with the small number of ART centers in the country, the expensive cost of treatment, and the lack of support mechanisms, only a few women may avail of such. According to a study, financial constraints are the first reason couples do not seek treatment (Flores, 2016). This may shock those in developed countries, but to reiterate, no public or private health insurance covers the costs of IVF treatments (Having a Baby in the Philippines? Hospital, Midwife, Delivery and IVF Costs, 2018). The Philippine Health Insurance Corporation or Philhealth, the government-owned corporation that provides insurance, so far, only covers “supervision of pregnancy with history of infertility” as a maternal comorbidities condition, with a total coverage of around $150. It is pretty telling that only wealthy Filipino celebrities have mostly benefited from more specialized ART services such as IVF and surrogacy (abroad) (Cabbuag, 2023; Tan, 2023). Notwithstanding the limits of ART services, the provider referral feature (possibly due to conscientious objection) that should supposedly be available to women is futile if they do not have the means.

Some might argue that there are some guidelines in the country. However, the Philippines has lagged in ART regulation compared to other developing nations. For instance, 20 years ago, Malaysia and Jordan had no guidelines concerning gamete donation (Chapter 8: Donation, 2007). At present, however, there have been developments in these countries. For instance, Jordan has some proposed laws up for discussion, and there have been studies that support the call for IVF regulation in Malaysia. One may also argue that, at least, IVF was not banned in the Philippines like in another predominantly Catholic country, Costa Rica. IVF was banned in Costa Rica from 2000 to 2015. Costa Rica, however, has an advanced healthcare infrastructure, and to catch up with the laws of more developed nations, Executive Decree No. 39210-MP-S was made to implement IVF to ensure the reproductive rights of people with infertility challenges (Valerio et al., 2017).

The lack of ART regulation also poses problems for the implications of such technology, particularly the practice of surrogacy. Since surrogacy is not “allowed” in the Philippines, vulnerable local women are hired to be surrogates for procedures performed abroad. Without clear laws, regulations, or prohibitions for such practices, Filipino women may be exposed to oppressive and exploitative situations. For instance, surrogate recruitment is done in “secret” through social media platforms (Sepe, 2019). Women who may be exploited by their recruiters often have no legal recourse available to them. In 2006, Senator Manny Villar filed Senate Bill No. 2344 Or The Act Prohibiting Surrogate Motherhood Including Infant Selling And Providing Penalties. The bill, however, never prospered. Indeed, many legal issues may arise due to the lack of regulation on surrogacy. The members of the PSRM even agree that having a law will protect all involved parties (Chiong, 2022).

## Conclusion

The lack of regulation of ART in the Philippines affects women, especially those who need more sophisticated medical interventions and psycho-social and emotional support. While some guidelines exist in regulating such technologies, relying on personal and moral motivations for the reproductive well being of women goes against their rights to appropriate treatment, reproductive autonomy, and essential medical healthcare. With the development of more advanced technology, Philippine law and policymakers must acknowledge the existence of ART and the lack of its regulation. This would ensure that the health needs of women with fertility issues would be addressed, their rights are protected and upheld, and they have the freedom to make reproductive decisions free from discrimination.

## Data Availability

The original contributions presented in the study are included in the article/supplementary material, further inquiries can be directed to the corresponding author.

## References

[B1] Abad P. J. Tumulak M. J. Guerbo R. Castro-Hamoy L. Bautista N. G. Nuique R.. (2023). Landscape of genetic counseling in the Philippines. J. Genet. Couns. 33, 934–942. 10.1002/jgc4.1804 37877196

[B2] Aguilar A. S. Novero V. J. Ong-Jao E. T. Utulo M. Cortes-Gaspar T. A. P. Enriquez-Gamboa M.. (2024). Agreement with “The ethical guidelines on the provision and practice of advanced reproductive technology and intrauterine insemination 2023” by the Philippine Society of Reproductive Medicine using online Delphi technique. Glob. Reprod. Health 9:e0091. 10.1097/GRH.000000000000009

[B3] Baron D. P. (2010). Morally motivated self-regulation. Am. Econ. Rev. 100, 1299–1329. 10.1257/aer.100.4.1299

[B4] Biana H. T. Dacela M. A. Domingo R. (2022). Disrupting Epistemic Injustice: Gender Equality and Progressive Philippine Catholic Communities. Available online at: https://philpapers.org/rec/BIADEI-2

[B5] Biana H. T. Domingo R. (2021). Lesbian single parents: reviewing philippine covid-19 policies. J. Int. Womens Stud. 22, 135–147.

[B6] Cabbuag S. I. (2023). “The road to visibility: IVF and motherhood journey of filipino Influencers,” in Resilience and Familism: The Dynamic Nature of Families in the Philippines, Contemporary Perspectives in Family Research, eds. V. L. Gregorio, C. M. Batan, and S. L. Blair (Leeds: Emerald Publishing Limited), 21–34.

[B7] Cabral E. (2013). Reproductive health law in the Philippines. J. ASEAN Fed. Endocr. Soc. 28:26. 10.15605/jafes.028.01.06

[B8] Chapter 8: Donation (2007). Chapter 8: donation. Fertil. Steril. 87, S28–S32. 10.1016/j.fertnstert.2007.01.092

[B9] Chiong O. P. (2022). A moral analysis of *in vitro* fertilization in the Philippine context. Philippiniana Sacra 57, 491–526. 10.55997/3004pslvii174a4

[B10] Chiu Y. (2012). Fight for reproductive health bill grows in the Philippines. Lancet 380:98. 10.1016/S0140-6736(12)61162-3 22803207

[B11] Dickens B. M. (2006). Ethical misconduct by abuse of conscientious objection laws. Med. Law 25:513. 17078524

[B12] Dupont P. (2013). Life and death in the Philippines. Facts Views Vis. Obgyn. 5, 274–277. 24753955 PMC3987382

[B13] Ebrahimzadeh Z. S. Latifnejad R. R. Janghorban R. Mojtaba M. B. S. Amirian M. Allan H. T. (2019). Infertile couples' needs after unsuccessful fertility treatment: a qualitative study. J. Caring Sci. 8, 95–104. 10.15171/jcs.2019.014 31249819 PMC6589480

[B14] FAQs | Kato Repro Biotech Center (2025). Available online at: https://www.katoreprobiotechcenter.com/faqs/ (accessed February 5, 2025).

[B15] Flores R. L. (2016). Infertility in the Philippines and natural procreative (napro) technology: a commentary. Sch. Acad. J. Biosci. 4, 328–331. 10.36347/sajb.2016.v04i04.005

[B16] Forman D. L. (2011). When bad mothers make worse law: a critique of legislative limits on embryo transfer. U. Pa. J. Soc. Change 14:273.

[B17] Gameiro S. Boivin J. Peronace L. Verhaak C. M. (2012). Why do patients discontinue fertility treatment? A systematic review of reasons and predictors of discontinuation in fertility treatment. Hum. Reprod. Update 18, 652–669. 10.1093/humupd/dms031 22869759 PMC3461967

[B18] Gebhart M. B. Hines R. S. Penman A. Holland A. S. (2016). How do patient perceived determinants influence the decision-making process to accept or decline preimplantation genetic screening? Fertil. Steril. 105, 188–193. 10.1016/j.fertnstert.2015.09.022 26474735

[B19] Having a Baby in the Philippines? Hospital Midwife, Delivery and IVF Costs. (2018). Wise. Available online at: https://wise.com/us/blog/cost-of-having-a-baby-in-philippines (accessed July 27, 2018).

[B20] Hill J. A. (2020). Infertility Treatment Is Essential Medical Care by WHO. Fertility Centers of New England.

[B21] In-Vitro Fertilisation Laws and Regulations. (2021). Available online at: https://www.zicolaw.com/resources/publications/in-vitro-fertilisation-laws-and-regulations/ (accessed *July* 5, 2021).

[B22] Jain M. Singh M. (2025). Assisted Reproductive Technology (ART) Techniques, in StatPearls. Treasure Island, FL: StatPearls Publishing. 35015434

[B23] Kaplan D. A. (2024). “Catholic patients and reproductive healthcare,” in Cultural Responsiveness in Assisted Reproductive Technology: Best Practices for Clinics and Affiliated Providers, ed. D. A. Kaplan (Cham: Springer Nature Switzerland), 187–200.

[B24] Lofredo M. P. P. (2016). “From bioethical definitions to the setting of public policy by the court of law in the Philippines,” in The 12th Asia Pacific Conference (APC 12) and The 15th Asian Bioethics Conference (ABC 15), 329.

[B25] Murray-Nevill J. (2023). One in five workers undergoing fertility treatment have quit their job due to the way they were treated, with women's progression hit hardest. Totaljobs. Available online at: https://www.totaljobs.com/media-centre/one-in-five-workers-undergoing-fertility-treatment-have-quit-their-job-due-to-the-way-they-were-treated-with-womens-progression-hit-hardest (accessed September 14, 2023).

[B26] Nugent C. (2018). What it was like to grow up as the world's first test-tube baby. TIME. Available online at: https://time.com/5344145/louise-brown-test-tube-baby/ (accessed July 25, 2018).

[B27] Priest M. (1997). The privatization of regulation: five models of self-regulation. Ottawa Law Rev. 29:233.

[B28] Sepe F. G. Jr. (2019). WANTED: Surrogate Mothers. ABS-CBN News. Available online at: https://news.abs-cbn.com/specials/wanted-surrogate-mothers (accessed February 11, 2025).

[B29] Shirai C. (2021). The socio-cultural context of coping with infertility: a case study from three Asian countries. Asian Stud. 16, 1–9. 10.14945/00027888

[B30] Skinner F Clark B. (2021). How providing infertility support can foster an inclusive culture. Personnel Today (blog). Available online at: https://www.personneltoday.com/hr/infertility-support-for-employees-diversity-inclusion/ (accessed August 17, 2021).

[B31] Stulberg D. B. Hoffman Y. Dahlquist I. H. Freedman L. R. (2014). Tubal ligation in catholic hospitals: a qualitative study of ob–gyns' experiences. Contraception 90, 422–428. 10.1016/j.contraception.2014.04.015 24912729 PMC4154979

[B32] Taguibao A. J. Bance L. O. (2022). It's the climb: the reproductive journey and well-being of Filipino women with infertility. Philipp. Soc. Sci. J. 5, 28–43. 10.52006/main.v5i4.581

[B33] Tan F. M. Jr. (2023). Surrogacy in the Philippine context: bane or boon? Asian J. Soc. Sci. Stud. 5, 99–107. 10.34104/ajssls.023.0990107

[B34] The People Department (2023). Fertility Treatment Leave. Oxford: University of Oxford. Available online at: https://hr.admin.ox.ac.uk/fertilitytreatment-leave (accessed March 9, 2025).

[B35] Valerio C. Vargas K. Raventós H. (2017). IVF in Costa Rica. JBRA Assist. Reprod. 21, 366–369. 28985042 10.5935/1518-0557.20170060 PMC5714607

[B36] Van den Broeck U. Holvoet L. Enzlin E. Bakelants E. Demyttenaere K. D'Hooghe T. (2009). Reasons for dropout in infertility treatment. Gynecol. Obstet. Invest. 68, 58–64. 10.1159/000214839 19401627

[B37] Zuckerman S. Gooldin S. Zeevi D. A. Altarescu B. (2020). The decision-making process, experience, and perceptions of preimplantation genetic testing (PGT) users. J. Assist. Reprod. Genet. 37, 1903–1912. 10.1007/s10815-020-01840-4 32462417 PMC7468006

